# Psychophysiological Outcome Responses in Human Pavlovian Fear Conditioning: A Prediction Error Analysis

**DOI:** 10.1111/psyp.70300

**Published:** 2026-04-22

**Authors:** Huaiyu Liu, Josie Linnell, Dominik R. Bach

**Affiliations:** ^1^ Department of Imaging Neuroscience, UCL Queen Square Institute of Neurology University College London London UK; ^2^ Transdisciplinary Research Area Life and Health, Centre for Artificial Intelligence and Neuroscience; and Institute of Computer Science University of Bonn Bonn Germany; ^3^ Department of Psychiatry and Psychotherapy, and Institute of Experimental Epileptology and Cognition Research University Hospital Bonn, University of Bonn Bonn Germany

**Keywords:** associative learning, aversive prediction errors, Pavlovian fear conditioning, psychophysiological measure

## Abstract

Prediction errors (PE) are thought to drive associative learning. While neural signals consistent with PE encoding have been identified, the expression of PE in psychophysiological indices remains debated. Here, we sought to fill this gap by investigating responses to unconditioned stimulus (US) occurrence and probability in skin conductance responses (SCR), pupil size responses (PSR), heart period responses (HPR), and respiration amplitude responses (RAR). Data set 1 consisted of eight published studies (N_1_ = 264) using differential fear conditioning with partial reinforcement (50%), and novel data set 2 (*N*_2_ = 29) parametrically varied US probability (20%/50%/80%). Across both data sets, all modalities showed differential responses to the US compared to US omission. In data set 1, there was evidence for responses to unexpected as compared to expected US omission in all modalities, but no responses were consistent with signed or unsigned PE encoding. Similarly, data set 2 provided no evidence that US or US omission responses monotonically related to outcome probability, which is incompatible with both signed and unsigned PE encoding. In conclusion, all recorded psychophysiological signals responded strongly to US and less strongly to unexpected US omission, with no evidence of either signed or unsigned PE encoding.

## Impact Statement

Pavlovian reward learning is driven by prediction errors (PE), but it remains unclear whether this is also the case for aversive learning, and to what extent PE are expressed in physiological indices. Here, we examined responses to outcome magnitude and probability to assess the expression of PE across four psychophysiological indices (SCR, PSR, HPR, RAR). Results replicated the well‐known US response in all modalities; however, we found clear evidence against signed PE encoding and no evidence for unsigned PE encoding. These findings may inform future work on computational modeling of fear learning and reconsolidation‐based clinical interventions.

## 1 Introduction

Learning to detect, respond, and predict potential threats in the environment is important for most animals and humans (Brochard et al. 2025). However, when fear and avoidance become exaggerated or persist in safe contexts, they can contribute to the development and persistence of clinical conditions such as anxiety disorders (Craske et al. 2017). Understanding the mechanisms underlying fear learning could be crucial for advancing clinical treatments for pathological fear (Beckers et al. 2023). A canonical paradigm to study this in the laboratory is Pavlovian fear conditioning (Maren 2001; Pavlov 1927; Watson and Rayner 1920), where an initially neutral stimulus (conditioned stimulus, CS) is repeatedly paired with a naturally aversive stimulus (unconditioned stimulus, US), and subsequently comes to elicit behavioral or physiological responses (conditioned response, CR).

One dominant assumption is that learning is driven by prediction errors (PE), which reflect the signed difference between predicted and actual outcomes (Rescorla 1972). In addition to signed PE, some learning theories posit a contribution of unsigned PE signals, sometimes referred to as surprise (Pearce and Hall 1980), which reflect the absolute difference, or the magnitude of the mismatch, between predicted and actual outcomes. While neural substrates of PE encoding have been widely demonstrated during maintenance of Pavlovian reward associations (Schultz 2016; Schultz et al. 1997), there are rather more sparse reports for Pavlovian fear conditioning (Gorka et al. 2023; Ojala et al. 2022; Yau and McNally 2018), which is known to rely on a neural circuit different from that supporting reward learning (LeDoux 2000). Alternatively, it has been suggested that learning might be driven by other types of neural or computational quantities. For example, in distributional reinforcement learning (Dabney et al. 2020), different neurons make different predictions, such that PE differs across neurons. For another example, an organism can learn the likelihood of observed outcomes without encoding PE (Tzovara et al. 2018). As such, the investigation of PE encoding is of relevance for the advancement of algorithmic learning theories.

Moreover, PE encoding might also be more directly relevant for clinical purposes. For example, it has been suggested that memory can be destabilized by re‐activation, and its re‐stabilization can be suppressed by pharmacological intervention (Nader et al. 2000; see for review e.g., Schroyens et al. 2023). This could potentially be leveraged to treat phobias or posttraumatic stress disorder (Kindt 2018; Kindt et al. 2009). There is some evidence that this memory destabilization is driven by PE during re‐activation (Sinclair and Barense 2018), potentially reflecting either signed PE or unsigned surprise signals. Thus, clinical interventions based on reconsolidation theory might benefit from an online assessment of whether PE was elicited.

Psychophysiological responses, in particular those elicited via the autonomic nervous system, provide an accessible window into associative learning processes. CR are often thought to reflect learning quantities (Ojala and Bach 2020). In turn, unconditioned responses elicited by the US can be modulated by the preceding CS (Kimble and Ost 1961). For example, US‐elicited SCR appear to be smaller when the US is signaled by a CS+, compared to a no‐CS baseline (Knight et al. 2010; Redondo et al. 2015). Relatedly, when the CS is partially reinforced, SCR after US omission have been observed (Spoormaker et al. 2012; Stemerding et al. 2022, but see Bach and Friston 2012). Both of these observations are compatible with signed PE encoding. Other observations of US response modulation are only partly compatible with this account. For example, PSR appeared greater after high versus low surprise stimuli (Braem et al. 2015; Browning et al. 2015; O'Reilly et al. 2013), where surprise is an unsigned PE‐related quantity. Other studies have reported increased or decreased pupil size under high versus low uncertainty (Lavin et al. 2014; Satterthwaite et al. 2007). In addition, decreased heart rate (i.e., increased HPR) has been observed following sensorimotor‐related error trials compared to correct trials (Schlerf et al. 2012). Another study reported a faster and more prolonged heart rate deceleration in response to punishment feedback as opposed to reward feedback (Kastner et al. 2017).

Two key gaps emerge from this literature. First, there is a lack of studies systematically assessing different psychophysiological indices within the same paradigm. Second, there is no systematic assessment of the extent to which the different quantities assessed in previous work are compatible with signed or unsigned PE encoding in the sense of formal learning theories.

To address these gaps, the present work investigated SCR, PSR, and HPR as in previous literature, and additionally included RAR for exploration. We used axiomatic tests (see Methods) to systematically evaluate the compatibility of empirical results with both signed and unsigned PE encoding (Caplin and Dean 2008). We combined eight previously published studies into data set 1 to maximise statistical power. In parallel, we conducted a dedicated study, data set 2, where we parametrically varied US probabilities to allow a more nuanced examination.

## 2 Methods

### 2.1 Participants

Data set 1 comprised eight previously published fear conditioning studies from the group of the last author, with a combined sample size of 264 participants (see Table 1 for details). Data set 2 (*N* = 29) is first published here (Table 1). For all studies, healthy participants were recruited from the student and general population. Each study, including the procedure of obtaining written informed consent, was conducted in accordance with the Declaration of Helsinki and approved by a research ethics committee (Data set 1: Kantonale Ethikkommission Zurich, KEK‐ZH‐2013‐0118; Data set 2: UCL REC 6649/005). See Table 1 for demographics and general information.

**TABLE 1:** Overview and demographics for the nine included studies.

	Data modalities	Study code	*N*	Self‐reported sex (female/male)	Age (SD)	Numer of CS+ trials	Number of CS− trials	CS type
Dataset 1	SCR PSR HPR RAR	FER01	30	18/12	23.9 (4.4)	32	10	Visual
		FER02	72	39/33	24.2 (3.9)	32	10	Visual
		PubFe	22	15/7	26.4 (5.2)	80	80	Auditory
		SC4B	21	11/10	22.9 (3.0)	96	96	Auditory
	SCR HPR RAR	DoxMemP	20	13/7	26.1 (4.1)	80	80	Visual
		FR	31	23/8	23.3 (3.6)	80	80	
	SCR PSR	FSS6B	18	7/11	25.7 (5.0)	48	48	Somatosensory
		VC7B	21	15/6	27.9 (5.5)	80	80	Visual
Dataset 2	SCR PSR HPR RAR	PCF3	29	14/15	30.7 (10.5)	68	67	Visual

Abbreviations: HPR, heart period responses; PSR, pupil size responses; RAR, respiration amplitude responses; SCR, skin conductance responses.

To put the sample size of data set 2 into context, we conducted an approximative bootstrapping power analysis for SCR. Our approach of cluster‐based permutation testing makes no a priori assumptions on where an effect occurs within a time interval of interest. Thus, rather than assuming an effect size (which neglects temporal variability of the response), we assumed a minimal interesting effect magnitude of 20% peak difference between any two conditions. To simulate the variability of response latencies within this time window, we bootstrapped responses from four independent data sets containing SCR evoked by noxious electrical stimulations collected in our laboratory (SCRV6, https://doi.org/10.5281/zenodo.4287271; RRM1‐2, https://doi.org/10.5281/zenodo.4287111; and SPRM, https://doi.org/10.5281/zenodo.10848890). Thus, we randomly sampled data for 29 participants with two within‐subject conditions, assuming a peak difference of 20%. We then applied the same cluster‐based permutation test used for data set 2 to this simulated data set, using an identical SCR response window (i.e., 2–10 s after US onset). This bootstrapping procedure was repeated 30 times. A significant condition difference was found in each simulation, thus suggesting a power of larger than 95% at this effect magnitude.

### 2.2 Stimuli and Experimental Procedure

All nine studies used a delay fear conditioning procedure. Data set 1 used differential conditioning with one partially (50%) reinforced CS+, and one never‐reinforced CS−. Data set 2 used three CS+ with different reinforcement rates (20%/50%/80%), and no CS−. In previously published data set 1, CS lasted 4 s, and US onset was after 3.5 s. In data set 2, timings were optimized for the analysis of US response modulation, and CS lasted 6.5 s, while US onset was after 6 s. For both data sets, US duration was 0.5 s, and the inter‐trial‐interval (ITI) was randomly drawn from an interval of 7–11 s.

Data set 1 used three types of CS (visual, auditory, and somatosensory; Table 1), and data set 2 used visual CS only. The US was a train of electric simulations delivered by a current stimulator (Digitimer DS7A, Welwyn Garden City, UK) and a ring‐pin electrode. For each participant, US intensity was determined using a two‐step algorithm: a clearly painful intensity was identified using an ascending staircase procedure, which was then followed by delivering 14 random stimuli below this upper limit. The final US intensity was determined such as to elicit a discomfort rating of 85% compared to a clearly painful stimulus.

The number of CS+/CS− trials is listed in Table 1. Some studies in data set 1 included two distinct CS+ with the same reinforcement rate (FER01/02, SC4B, FSS6B, VC7B), and some also included two CS− (SC4B, FSS6B, VC7B). For studies FER01/02, both CS+ belonged to the same category (triangles of varying colors). For SC4B, FSS6B, and VC7B, the different CS+ and CS− differed in perceptual complexity but were within the same sensory modality. Previous studies found comparable learning between the two CS sets (Staib et al. 2020; Staib and Bach 2018). Hence, to enhance the signal‐to‐noise ratio, we collapsed the responses across multiple CS conditions based on the assumption that learning differences between the two CS sets would be negligible for the present purpose.

### 2.3 Data Recording

For data set 1, pupil diameter and gaze direction were measured using an EyeLink 1000 System (SR Research, Ottawa, ON, Canada) with a sampling rate of 500 Hz. Gaze calibration was conducted using the nine‐point calibration method provided by the EyeLink 1000 software. Participants rested their heads on a chin rest, positioned 70 cm away from the monitor (Dell P2012H, 20″ display, 5:4 aspect ratio, 60‐Hz refresh rate, the width and height of the screen were 44.2 and 24.9 cm, respectively). For the other three modalities, the output signals were digitized at a 1000 Hz sampling rate using a DI‐149 ad converter (Dataq Inc., Akron, OH, USA) and recorded with Windaq software (Dataq Inc.). SCR electrodes were positioned on the thenar and hypothenar regions of the left hand for studies FER01/02, and on the non‐dominant hand for all other studies. We used 8‐mm Ag/AgCl cup electrodes (EL258, Biopac Systems Inc., Goleta, CA, USA) with 0.5% NaCl gel (GEL101, Biopac Systems Inc., Goleta, CA, USA; Hygge and Hugdahl 1985). The skin conductance signal was amplified using an SCR coupler/amplifier (V71‐23, Coulbourn Instruments, Whitehall, PA, USA). Electrocardiogram (ECG) signals were recorded using four 45‐mm pre‐gelled Ag/AgCl adhesive electrodes, which were placed on the four limbs. The experimenter visually determined the lead configuration (I, II, III) or augmented lead (aVR, aVL, aVF) that exhibited the most prominent R spike and selected this configuration for recording. The data were pre‐amplified and processed with a 50‐Hz notch filter using a Coulbourn isolated five‐lead amplifier (LabLinc V75‐11, Coulbourn Instruments, Whitehall, PA). Respiratory data were recorded using an aneroid chest bellows (V94‐19, Coulbourn Instruments, Whitehall, PA, USA) in combination with a differential aneroid pressure transducer (V9415, Coulbourn), positioned around the rib cage at the lower sternum. The signal was then amplified via a resistive bridge strain gauge transducer coupler (V72‐25B, Coulbourn).

For data set 2, pupil diameter and gaze direction were measured using the same EyeLink 1000 system as in data set 1, but with different monitor distance settings: participants were positioned 64.5 cm away from the monitor, the width and height of the screen were 31.2 and 22.7 cm, respectively, and the distance from the EyeLink to participants' eyes was 48.5 cm. Skin conductance was recorded with a custom‐built coupler on the thenar/hypothenar of the non‐dominant hand using 8 mm Ag/AgCl cup electrodes (EL258, Biopac Systems., Goleta CA, USA) and 0.5%‐NaCl electrode paste (GEL101; Biopac Systems). Heartbeat timestamps and respiratory data were recorded using a pulse oximeter (8600, Nonin, Plymouth MN, USA) with a fiber optic sensor and a respiratory belt with a custom‐built transducer. All signals were amplified and digitized using a CED Micro1401 interface (Cambridge Electronic Design, Cambridge, UK) and recorded with Spike2 software (Cambridge Electronic Design).

### 2.4 Data Preprocessing

Data preprocessing was conducted in MATLAB (version R2019a, MathWorks, Natick, MA, USA) and PsPM (Psychophysiological Modeling, https://bachlab.github.io/PsPM/, version 6.1.2), a MATLAB toolbox designed for preprocessing and modeling psychophysiological data (Bach et al. 2018; Bach and Melinscak 2020).

For eye‐tracking data in both data sets, we averaged gaze direction from both eyes (if available) and excluded any time bins from analysis during which gaze direction deviated beyond ±5° visual angle from the fixation point, following the approach used in previous work (Korn et al. 2017). Next, pupil size data were preprocessed following an established procedure (Kret and Sjak‐Shie 2019) as implemented in PsPM, which included identification of valid samples by range, speed, edge, trendline, and isolated sample filtering. When data from both eyes were available, they were averaged, and any missing data points were linearly interpolated. Pupil data were processed using a low‐pass Butterworth filter with a 50 Hz cutoff and downsampled to 10 Hz.

For both data sets, SCR artifacts were identified through an automatic quality assessment, which excluded data outside the range of 0.05–60 μS or with a slope larger than 10 μS s^−1^, followed by visual inspection. Artifact periods were linearly interpolated for filtering and visualization and excluded for statistical tests. SCR data were then processed using a first‐order bidirectional low‐pass Butterworth filter (5 Hz) and downsampled to 10 Hz (Bach et al. 2010; Staib et al. 2015). No high‐pass filtering was applied. Next, SCR was z‐transformed to account for between‐subjects variance in SCR amplitude, which might be due to peripheral factors such as skin properties (Bach et al. 2010).

For data set 1, QRS complexes were identified from the ECG data using a modified version of the Pan and Tompkins algorithm (Paulus et al. 2016) to generate heartbeat timestamps. For data set 2, the pulse waveform was processed by the pulse oximeter. For both data sets, heartbeat timestamps were then converted into an interpolated heart period signal at 10 Hz resampling frequency; heart period values outside of the range 0.6–1.5 s (corresponding to 40–100 bpm) were removed and linearly interpolated.

For both data sets, raw respiratory signals were processed using a previously established respiratory cycle detection algorithm (Bach et al. 2016) and converted into an interpolated respiration amplitude time series with a sampling rate of 10 Hz.

### 2.5 Statistical Analyses

#### 2.5.1 Axiomatic Tests

**FIGURE 1:**
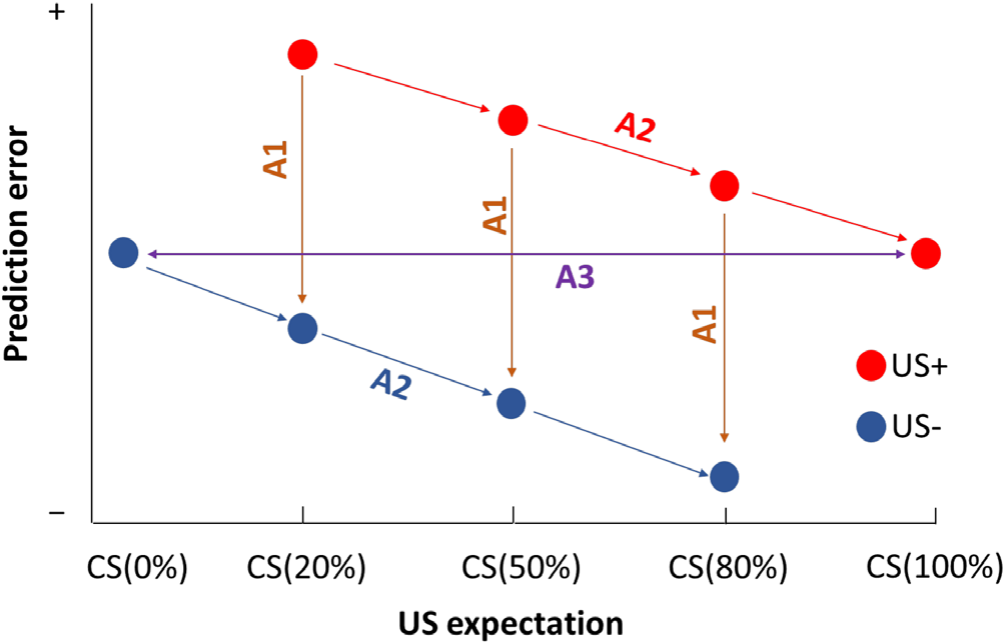
A diagram of necessary and sufficient conditions for signed prediction errors (PE), adjusted from (Ojala et al. 2022). Lines depict the tested contrasts, which were tested either all in the direction of the arrows, or all into the opposite direction. See main text for details of each axiom.

Some previous neuroimaging work has performed a linear regression of presumed PE indices in the data onto PE computed in a computational model. There are two reasons why we use a different approach. First, where a relation between neural signals and signed PE has been found, this relation is highly non‐linear (Schultz 2016), and therefore a linear regression analysis of the data onto estimated PE is likely to be inadequate (Caplin and Dean 2008). Second, model‐based approaches that map physiological signals to trial‐by‐trial outputs from a predefined learning algorithm require strong assumptions about the form and dynamics of the learning process. In addition, different learning models and parameterizations can yield highly correlated but quantitatively distinct PE estimates, which complicates interpretation. This is why we opted for an axiomatic approach. This allows testing whether physiological signals are compatible with PE encoding in a model‐agnostic manner, without committing to any specific learning rules or biophysical mappings. Specifically, if signed PE monotonically (linearly or non‐linearly) maps onto a physiological signal, then this signal must fulfill three axioms (Caplin and Dean 2008), which are visualized (for a linear mapping) in Figure 1 (panel A). In turn, these axioms constitute sufficient and necessary conditions for verifying the expression of signed PE in a physiological signal. Axiom 1 (A1): When fixing the probability of receiving the US, a greater US magnitude should elicit a higher/lower physiological signal. Axiom 2 (A2): When fixing the US magnitude, a lower probability of receiving the US should result in a higher/lower PE signal. Here, higher/lower should be read as either higher for both Axioms 1 and 2, or lower for both. Axiom 3 (A3): Regardless of US type, if the US is fully predicted, there should be no PE signal.

In addition, the expression of unsigned PE (i.e., surprise) would follow a different set of theoretical conditions, as shown in Supporting Information (Figure S1). Condition 1 (C1): When fixing the probability of receiving the US, the physiological responses should be higher when the outcome is more unexpected. Condition 2 (C2, corollary of C1): Comparable physiological responses for both outcomes should occur when the US probability is 50%. Condition 3 (C3): When fixing US magnitude, a 20% probability of receiving the US should elicit the highest unsigned PE signal for US delivery trials, compared with 50% and 80% probabilities, with the opposite pattern expected for US omission trials. Condition 4 (C4): This condition is identical to the aforementioned Axiom 3 defined for signed PE.

In the present work, we examined A1 and A2 in both data sets (see Table 2 for details) using all trials, assuming that participants rapidly learned the CS–US contingencies. A1 was examined by comparing US presentation (US+) trials and US omission (US−) trials for the same CS+, that is, when fixing the expected US probability. A2 was examined by comparing US omission trials with different probabilities (data sets 1 and 2), as well as US presentation trials with different probabilities (data set 2). A3 was not examined, as none of the data sets contained a fully reinforced CS+ condition (i.e., US+ (100%)), preventing a direct comparison with CS− (i.e., US− (0%)) trials.

**TABLE 2:** Axiomatic tests and corresponding linear mixed‐effects model syntax.

Data set	Axiomatic test 1	Axiomatic test 2	Model syntax
1	US+ (50%) versus US− (50%)	US− (0%) versus US− (50%)	lmer(DV ~ condition + (1 | study/ppid))
2	US+ (20%) versus US− (20%)	US+ (20%) versus US+ (50%)	lmer(DV ~ condition + (1 | ppid))
	US+ (50%) versus US− (50%)	US+ (20%) versus US+ (80%)	
	US+ (80%) versus US− (80%)	US+ (50%) versus US+ (80%)	
		US− (20%) versus US− (50%)	
		US− (20%) versus US− (80%)	
		US− (50%) versus US− (80%)	

*Note:* In the column Model syntax, DV refers to psychophysiological response (SCR/PSR/HPR/RAR), study refers to the grouping variable study and ppid refers to participants nested within each study, lmer is an R function for fitting linear mixed‐effects models from the R package lme4 (version 1.1.31).

All results reported in the main text refer to the entire set of trials, assuming rapid learning and a near‐constant US prediction during the experiment. Since this could be considered too strict an assumption, we repeated all analyses including only trials from the second half of each experiment. The results were highly similar to those obtained using the full data sets (see Supporting Information).

#### 2.5.2 Cluster‐Based Permutation Tests

For data set 1, we first combined data from studies that recorded the same modalities to enhance the signal‐to‐noise ratio. For both data sets, we then baseline‐corrected the data by subtracting the average of a 0.5 s pre‐US interval per participant. Next, for each modality, we examined A1 and A2 using linear mixed‐effects models (see Table 2 for details) for each 0.1‐s time bin over the response interval for SCR/PSR/HPR/RAR. For each modality, we selected a response interval that is likely to contain the peak of a US‐elicited response based on previous work. Specifically, for SCR the response interval was 2–10 s after US onset (Bach et al. 2010), for HPR and RAR the response interval was 0–10 s after US onset (Bach et al. 2016; Paulus et al. 2016), and for PSR the response interval was 0–4 s after US onset (Korn et al. 2017; Mathôt and Vilotijević 2023).

Our selected response intervals contained hundreds of time bins, posing a substantial multiple comparison problem (Saville 1990). To account for this, we used cluster‐based permutation tests (Maris and Oostenveld 2007). This method identifies temporally contiguous intervals (“clusters”) of above‐threshold effects and assesses their combined test statistics by comparing them to a null distribution generated through random permutations of the condition labels. A cluster is considered significant if its test statistic exceeds the critical threshold derived from the null hypothesis distribution. By analyzing clusters rather than isolated time points, this method increases statistical power while preserving the family‐wise error of false positives, compared to time‐point wise corrections (such as the Holm‐Bonferroni correction). We used a time‐bin inclusion threshold of *p* < 0.05 (Maris and Oostenveld 2007). For each axiomatic test for each modality, we performed an identical cluster‐based permutation test as in previous work (Maris and Oostenveld 2007). See Table 2 for details of examining A1 and A2. Since the axiomatic analysis only allows interpreting conjunctions of significant tests (rather than individual *p*‐values), there was no multiple comparison problem across tests.

#### 2.5.3 Peak‐Scoring Analyses

As a robustness analysis for the null findings relating to US probability in data set 2, we conducted a peak‐scoring analysis for SCR and PSR, for which established analysis algorithms exist. For SCR, the response onset window was 1–4 s after US onset, and the peak window was 0.5–5 s after the SCR onset. The response amplitude was then calculated by subtracting the onset amplitude from the peak amplitude (Boucsein 2012). For PSR, the peak window was 1–4 s after US onset, while the baseline response was defined as the average PSR 1–0 s prior to US onset. The response amplitude was calculated by subtracting this baseline response from the maximum PSR observed within the peak window (Steinhauer et al. 2022).

Based on the extracted peak‐scored responses, we performed paired *t*‐tests to examine A1 and A2 for SCR and PSR, respectively.

### 2.6 Data and Code Availability

All data are publicly available on Zenodo (Bach and Sporrer 2024; Khemka et al. 2021; Korn et al. 2021; Staib et al. 2021, 2021a, 2021b; Tzovara et al. 2021; Zimmermann et al. 2021a, 2021b; https://zenodo.org/communities/pspm/, see reference list for study‐specific URLs). Anonymized pre‐processed data for both data sets, as well as scripts for data analysis and Supporting Information are available on OSF (https://osf.io/5tj79/).

## 3 Results

### 3.1 Data Set 1

Data set 1 included two US probabilities: 0% (CS− trials) and 50% (CS+ (50%)/CS− (50%) trials).

#### 3.1.1 Response to Outcome Magnitude

**FIGURE 2:**
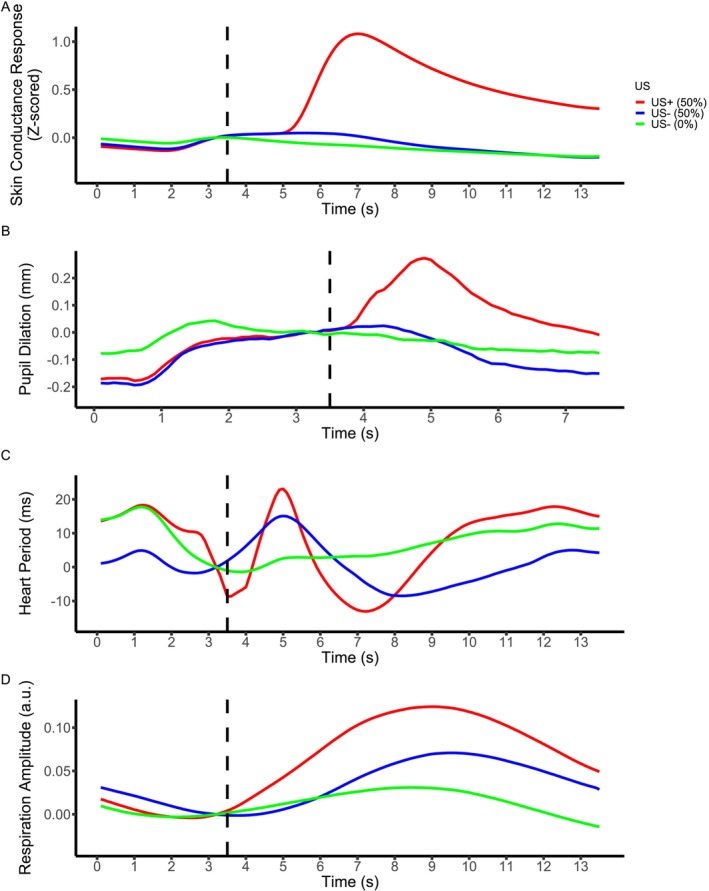
Linearly interpolated data, for each modality, collapsed across all trials and participants in data set 1. Data were baseline‐corrected by subtracting the average of a 0.5 s pre‐US interval per participant. Note that linearly interpolated data were used for visualization purposes only, and all statistical analyses were conducted using the non‐interpolated data. Panels A–D show SCR, PSR, HPR, and RAR under three conditions: US− (0%) (green), US+ (50%) (red), and US− (50%) (blue). See Table 3 for details on significant clusters identified over time.

To test the effect of outcome magnitude, we compared responses to US (US+) and to US omission (US−) on CS+ trials (Table 3). We observed differential responses in all data modalities. Responses to US were larger than to US omission in SCR, PSR and RAR, whereas in HPR, we observed smaller responses to US, that is, tachycardia. Figure 2 displays the locations of the significant clusters in time for each modality and Table 3 lists details of these significant clusters.

**TABLE 3:** Results of cluster‐level permutation tests for SCR, PSR, HPR, and RAR in data set 1.

Axiom	SCR (*z*‐score)	PSR	HPR	RAR
A1	**Result:** US+ (50%) > US− (50%), *p* < 0.001, [5.5 s, 13.5 s]	**Result:** US+ (50%) > US− (50%), *p* < 0.001, [4.0 s, 7.5 s]	**Results:** US+ (50%) > US− (50%), *p* < 0.001, [8.4 s, 13.5 s]; US+ (50%) < US− (50%), *p* < 0.001, [3.5 s, 4.3 s]; US+ (50%) < US− (50%), *p* < 0.001, [5.7 s, 7.7 s]	**Result:** US+ (50%) > US− (50%), *p* < 0.001, [3.5 s, 13.5 s]
A2	**Result:** US− < US− (50%), *p* < 0.001, [5.5 s, 13.5 s]	**Results**: US− < US− (50%), *p* < 0.001, [3.5 s, 4.5 s]; US− > US− (50%), *p* < 0.001, [5.7 s, 7.5 s]	**Results**: US− > US− (50%), *p* < 0.001, [7.0 s, 13.5 s]; US− < US− (50%), *p* < 0.001, [3.5 s, 6.3 s]	**Result:** US− < US− (50%), *p* < 0.001, [6.8 s, 13.5 s]

*Note:* *Z*‐score is computed for SCR only. All modalities are baseline‐corrected by subtracting the average of a 0.5 s pre‐US interval per subject.

#### 3.1.2 Response to Outcome Probability

To test the effect of outcome probability, we compared responses to US omission on non‐reinforced CS+ and CS− trials (Figure 2). We observed differential responses in all data modalities. Responses on CS− trials were smaller than those in non‐reinforced CS+ trials in SCR and RAR. PSR was initially smaller and then larger on CS− trials. The biphasic HPR was initially smaller on CS− trials and then larger. Figure 2 displays the locations of the significant clusters in time for each modality and Table 3 lists details of these significant clusters.

#### 3.1.3 Axiomatic Analysis

All modalities showed an effect of outcome magnitude and outcome probability, such that we analyzed whether their direction conformed to the axioms for signed PE encoding. Specifically, if larger US magnitude (i.e., a worse outcome than expected) elicits larger responses (A1), then more unexpected US omission on CS+ trials (i.e., a better outcome than expected) should elicit smaller responses (A2). On the contrary, if larger US magnitude elicits smaller responses, then more unexpected US omission on CS+ trials should elicit larger responses. Following this logic, the direction of responses for SCR and RAR was incompatible across A1 and A2. For PSR, statistical tests for A2 indicated both a larger and a smaller response on CS+ trials. When considering the entire time course from CS onset, pupil size on all trial types appeared to return to the same baseline with no appreciable late US omission response, suggesting that the early larger PSR reflect a US omission response. Similarly, for HPR the biphasic response seemed to be clearly larger for CS+ than CS− trials. Thus, these modalities again offered no evidence for signed PE encoding across A1 and A2.

For unsigned PE encoding, when US prediction was 50%, both US and US omission trials should elicit comparable psychophysiological responses (C2). This was evidently not the case in either data set, as US responses were consistently higher than US omission responses on these trials.

### 3.2 Data Set 2

Data set 2 included three different US probabilities: 20% (CS+ (20%)/CS− (20%) trials), 50% (CS+ (50%)/CS− (50%) trials), and 80% (CS+ (80%)/CS− (80%) trials).

#### 3.2.1 Response to Outcome Magnitude

**FIGURE 3:**
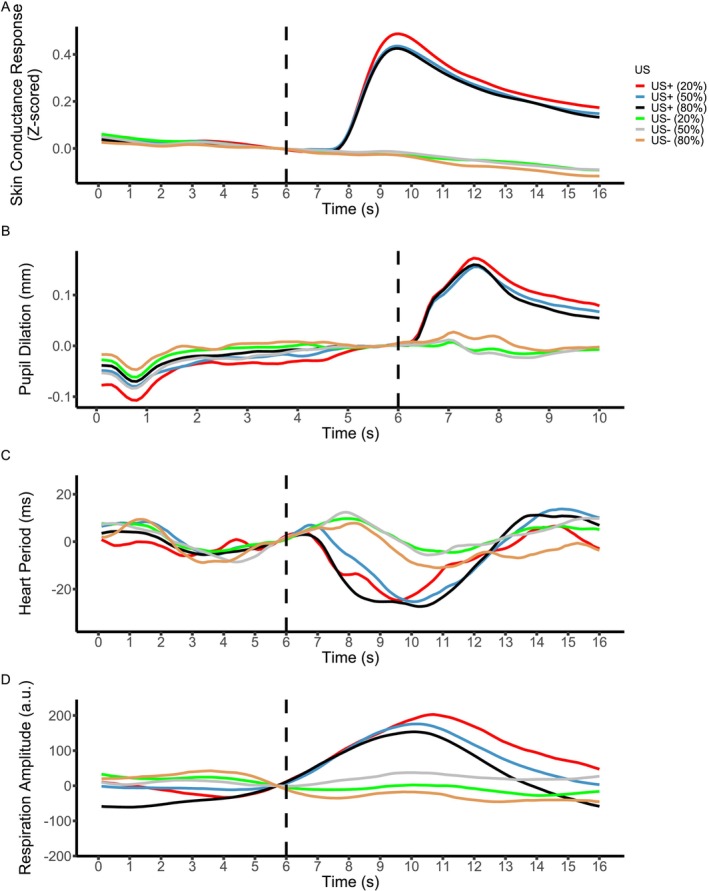
Linearly interpolated data, for each modality, collapsed across all trials and participants in data set 2. Data were baseline‐corrected by subtracting the average of a 0.5 s pre‐US interval per participant. Note that linearly interpolated data were used for visualization purposes only, and all statistical analyses were conducted using the non‐interpolated data. Panels A–D show SCR, PSR, HPR, and RAR during US presentation trials with three varying probabilities 20%/50%/80%, as well as during US omission trials with the same probabilities, respectively. See Table 4 for details on significant clusters identified over time.

To test the effect of outcome magnitude, we compared responses to US presence and to US omission trials (Figure 3) for each of the three CS. We observed differential responses in all data modalities. Responses to US were larger than to US omission in SCR, PSR, and RAR, whereas in HPR, we observed smaller responses to US. Figure 3 displays the locations of the significant clusters in time for each modality and Table 4 lists details for these clusters. Similar results were found for SCR and PSR in the peak‐scoring analysis (see Supporting Information).

**TABLE 4:** Results of cluster‐level permutation tests for SCR, PSR, HPR, and RAR in dataset 2.

Axiom	SCR (*z*‐score)	PSR	HPR	RAR
A1	**Result:** US+ (20%) > US− (20%), *p* < 0.001, [8.0 s, 16.0 s]	**Result:** US+ (20%) > US− (20%), *p* < 0.001, [6.5 s, 10.0 s]	**Result:** US+ (20%) < US− (20%), *p* < 0.001, [7.2 s, 10.6 s]	**Result:** US+ (20%) > US− (20%), *p* < 0.001, [6.5 s, 13.5 s]
	**Result:** US+ (50%) > US− (50%), *p* < 0.001, [8.0 s, 16.0 s]	**Result:** US+ (50%) > US− (50%), *p* < 0.001, [6.5 s, 10.0 s]	**Result:** US+ (50%) < US− (50%), *p* < 0.001, [7.4 s, 11.9 s]	**Result:** US+ (50%) > US− (50%), *p* < 0.001, [6.0 s, 11.9 s]
	**Result:** US+ (80%) > US− (80%), *p* < 0.001, [8.0 s, 16.0 s]	**Result:** US+ (80%) > US− (80%), *p* < 0.001, [6.5 s, 10.0 s]	**Results**: US+ (80%) < US− (80%), *p* < 0.001, [7.3 s, 10.9 s]; US+ (80%) > US− (80%), *p* < 0.01, [13.2 s, 15.0 s]	**Result:** US+ (80%) > US− (80%), *p* < 0.001, [6.0 s, 12.2 s]
A2	No significant cluster identified	No significant cluster identified	**Result:** US+ (50%) > US+ (80%), *p* < 0.01, [7.3 s, 9.0 s]; No significant cluster identified for other contrasts	No significant cluster identified

*Note:* *Z*‐score is computed for SCR only. All modalities are baseline‐corrected by subtracting the average of a 0.5 s pre‐US interval per subject.

#### 3.2.2 Response to Outcome Probability

For SCR, PSR, and RAR, cluster‐based permutation tests revealed no significant differences in any of the comparisons. There was one significant cluster for HPR, indicating larger responses to US+ (50%) (compared to US+ (80%)) trials. Figure 3 displays the location of the significant cluster in time for HPR and Table 4 shows the details for this cluster. Peak‐scoring analyses did not reveal additional results (see Supporting Information).

#### 3.2.3 Axiomatic Analysis

For signed PE encoding, since no consistent effect of outcome probability was found in any of the modalities, there was no support for A2. The one significant cluster in HPR was not replicated in any other comparison for HPR. Descriptively, Figure 3 appears to suggest that more unexpected US led to larger responses for SCR, PSR, and RAR; however, this ordering was not apparent for more expected US omission. Overall, there was no support for signed PE encoding across axioms A1 and A2.

For unsigned PE encoding, C1 predicts larger responses for US than US omission trials in the 20% condition, and larger responses for US omission than US trials in the 80% condition. C2 predicts comparable responses for US and US omission trials in the 50% condition. However, results in the 50% and 80% conditions were inconsistent with both C1 and C2. According to C3, the 20% condition should elicit the largest responses on US trials, whereas the 80% condition should elicit the largest responses on US omission trials. Responses across all four modalities were incompatible with C3. Taken together, these findings provide no evidence for unsigned PE encoding when jointly considering C1–C3.

## 4 Discussion

Pavlovian fear conditioning is an important basic learning paradigm, but it remains unclear to what extent its learning is driven by signed or unsigned PE signals, as in Pavlovian reward learning. Here, we explored a potential expression of PE in different candidate psychophysiological responses (based on SCR, PSR, HPR, and RAR) in two independent data sets. We conducted cluster‐based permutation tests to examine responses to outcome magnitude and outcome probability.

Three main findings emerge. First, we observed the well‐known US response (compared to US omission) in all modalities and both data sets (Bach and Friston 2012). In a condition with 50% US, this finding is incompatible with an unsigned PE encoding. Second, we found either no evidence for response modulation by US probability (data set 2), or the direction of response modulation was incompatible with a signed PE encoding (data set 1). Third and relatedly, there was a response to unexpected US omission (non‐reinforced CS+ trials) in the same direction as the response to the US itself, compared to CS− trials in all modalities (data set 1), as has previously been observed for SCR (Spoormaker et al. 2012; Stemerding et al. 2022).

Crucially, in data set 1, while unexpected US omission responses have previously been termed “prediction error” signals, they do not conform to the notion of a signed prediction error as used in many computational learning models since Rescorla & Wagner (Miller et al. 1995; Rescorla 1972). A signed prediction error encoding implies that responses to better‐than‐expected outcomes should be in the opposite direction compared to responses to worse‐than‐expected outcomes. Since partially predicted US (compared to US omission, a worse‐than‐expected outcome) elicits larger SCR, PSR, and RAR, and smaller HPR, unexpected US omission (compared to expected US omission, a better‐than‐expected outcome) should elicit smaller SCR, PSR, and RAR, and larger HPR. The opposite, however, was the case in our data. This constitutes clear and statistically significant evidence against the notion of signed prediction error encoding in these psychophysiological signals. On a different note, although these US omission responses are in principle consistent with an unsigned PE encoding (Rouhani and Niv 2021), the fact that US response is larger than US omission response—even though both outcomes are equally unexpected at a 50% reinforcement rate—cannot be fully explained by unsigned PE magnitude alone. Furthermore, we note that the US omission response descriptively appears to occur earlier than the US response in SCR, PSR, and HPR. If reliable, this difference in response latency is also not predicted by unsigned PE encoding.

Data set 2 afforded more nuanced comparisons at different reinforcement rates. Importantly, both signed and unsigned prediction error encoding would imply an impact of reinforcement rate on responses. For signed PE encoding, the largest responses are expected at a 20% reinforcement rate for both US+ and US− trials. For unsigned PE encoding, the largest responses are expected at a 20% reinforcement rate for US+ trials and at an 80% reinforcement rate for US− trials. However, our results from data set 2 provide no evidence for either assumption. While the sample size was relatively modest, and clearer results might be found in a larger sample, we can clearly rule out any response of a magnitude that would allow single‐subject analysis, for example, for model fitting or clinical monitoring purposes.

One limitation of the present work is the absence of a fully predicted CS+ condition. Including this condition could provide an estimate of the US response and its habituation in a fully predictable context, in which signed and unsigned PE signals are minimal. This baseline would also allow assessing whether potential PE responses are superimposed onto a US response generated by a different mechanism. Future work may extend the experiment of data set 2 by adding a CS− and a fully reinforced CS+ condition, together with a larger sample size.

Importantly, our results do not imply that the neural system does not use PE encoding. PE encoding in peripheral indices, if it existed, should be considered an epiphenomenon. The presence of such an epiphenomenon would be of great interest, as it would allow a direct window into learning quantities. However, its absence does not afford conclusions about the neural system.

In sum, based on studies employing Pavlovian fear conditioning paradigms with electrical stimulations as the US, we identify the well‐known US response in all data modalities, replicate the previous finding of an unexpected US omission response in SCR, and extend it to all other investigated data modalities. At the same time, we find clear evidence against a signed PE encoding and no evidence for an unsigned PE encoding in this specific experimental protocol. Caution is warranted when making claims about PE encoding in paradigms involving other types of learning and/or outcome variables.

## Author Contributions

**Huaiyu Liu:** formal analysis, writing – original draft, writing – review and editing, visualization. **Josie Linnell:** writing – review and editing. **Dominik R. Bach:** data collection, data curation, conceptualization, methodology, writing – review and editing, supervision, funding acquisition, project administration.

## Funding

This work was supported by the Economic and Social Research Council (ES/W000776/1), Wellcome Trust (203147/Z/16/Z).

## Conflicts of Interest

The authors declare no conflicts of interest.

## Supporting information


**Table S1a:** Peak‐scoring analyses for SCR in data set 2: Paired *t*‐tests.
**Table S1b:** Peak‐scoring analyses for PSR in data set 2: Paired *t*‐tests.
**Table S2a:** Results of cluster‐level permutation tests for SCR, PSR, HPR, and RAR in data set 1 restricted to the second half of trials.
**Table S2b:** Results of cluster‐level permutation tests for SCR, PSR, HPR, and RAR in data set 2 restricted to the second half of trials.
**Table S3:** Results of cluster‐level permutation tests for SCR, PSR, HPR, and RAR in data set 1, restricted to A2, using models with baseline values as covariates.
**Figure S1:** A diagram of necessary and sufficient conditions for unsigned prediction errors (PE). Lines depict the tested contrasts, which were tested all in direction of the arrows.

## Data Availability

All data are publicly available on Zenodo (Bach and Sporrer 2024; Khemka et al. 2021; Korn et al. 2021; Staib et al. 2021, 2021a, 2021b; Tzovara et al. 2021; Zimmermann et al. 2021a, 2021b; https://zenodo.org/communities/pspm/, see reference list for study‐specific URLs). Anonymized pre‐processed data for both data sets, as well as scripts for data‐analysis and Supporting Information are available on OSF (https://osf.io/5tj79/).
